# Enhancing fear extinction

**DOI:** 10.7554/eLife.97633

**Published:** 2024-04-15

**Authors:** Sydney Trask, Nicole C Ferrara

**Affiliations:** 1 Department of Psychological Sciences, Purdue University West Lafayette West Lafayette United States; 2 Purdue Institute for Integrative Neuroscience, Purdue University West Lafayette West Lafayette United States; 3 Center for Neurobiology of Stress Resilience and Psychiatric Disorders, Rosalind Franklin University of Medicine and Science North Chicago United States; 4 Discipline of Physiology and Biophysics, Chicago Medical School, Rosalind Franklin University of Medicine and Science North Chicago United States

**Keywords:** memory, prediction error, extinction, Rat

## Abstract

Gradually reducing a source of fear during extinction treatments may weaken negative memories in the long term.

**Related research article** Kennedy NGW, Lee JC, Killcross S, Westbrook F, Holmes NM. 2024. Prediction error determines how memories are organized in the brain: a study of Pavlovian fear extinction in rats. *eLife* **13**:RP95849. doi: 10.7554/eLife.95849.

Memories of aversive or negative events are central to the survival of animals. However, fear learning and memory can become a challenge in people with certain conditions, such as specific phobias, anxiety or PTSD. Such individuals often avoid certain activities or situations, or start to transfer their fear to new stimuli. This avoidance strategy may help reduce fear in the short term, but but it can increase distress in the long term.

One method to alleviate fear is exposure therapy, in which individuals are subjected to objects or situations they are afraid of in a safe environment, without experiencing harm (Craske et al., 2018). For example, for someone with a fear of dogs, exposure-based therapy might include looking at pictures of dogs. This will initially produce a fear response that can include physiological changes, such as perspiration and elevated heart rate, and psychological changes, such as an increase in subjective fear. After several exposures to these stimuli, both physiological and psychological indices of fear will decline.

While this ‘extinction’ method is highly effective in minimizing fear in the short term, fear reduced through this method is highly susceptible to relapse. Even patients and subjects showing no signs of fear at the end of therapy are likely to experience a rebound after a certain amount of time or when they are re-exposed to the distressing stimulus (Bouton et al., 2021; Vervliet et al., 2013).

Decades of research suggests that this re-appearance of symptoms happens because extinction creates a new inhibitory memory that competes with – rather than updates – the original fear memory. Recent learning theories suggest that learned events are assigned a ‘prediction error’: if the prediction error is large (i.e., the new experience differs greatly from the past one) a new association will be formed. But if the prediction error is small (and the new experience is similar to the past one), the existing memory will be updated or overwritten with new information. Since fear- and anxiety-based disorders are on the rise, identifying intervention and treatment strategies to mitigate fear in a long term, relapse-resistant manner is therefore of upmost importance (Jacob et al., 2021; Siegel et al., 2022).

Now, in eLife, Nathan Holmes and colleagues – including Nicholas Kennedy as first author – report evidence that supports this recent learning theory (Kennedy et al., 2024). Kennedy et al. used a classical conditioning protocol in which they conditioned rats to fear a tone (conditional stimulus) by pairing its presentation with a brief electric shock applied to the foot (unconditional stimulus). Following this training, rats responded with freezing to the tone alone.

The researchers then used two different procedures to reduce the rats’ fear. The first group received a ‘standard’ extinction procedure during which the rats were exposed to several presentations of the sound only. The second group received a new ‘gradual’ extinction procedure, during which the tone was paired with a gradually weakened shock voltage: from 0.8 to 0.4, 0.2, and 0.1 mA. While both the traditional and gradual extinction lessened the fear response, the gradual procedure was able to also reduce relapse.

Relapse was tested at different time points following extinction treatment. When animals were re-exposed to the tone two weeks after the extinction procedures, the traditional group showed an increase in fear (e.g., more freezing) while the gradual extinction group showed less fear. The gradual extinction group was also less likely to relapse even when the animals received a brief foot shock without sound (known as ‘reinstatement’) after the extinction treatment. However, both groups were similarly susceptible to relapse if there was a change in environmental conditions from conditioning to extinction, for example, changing the physical chamber or waiting a long time between conditioning and extinction (Figure 1). Thus, the efficacy of gradual extinction relies on similar contextual parameters during conditioning and extinction to optimally minimize relapse.

**Figure 1.**
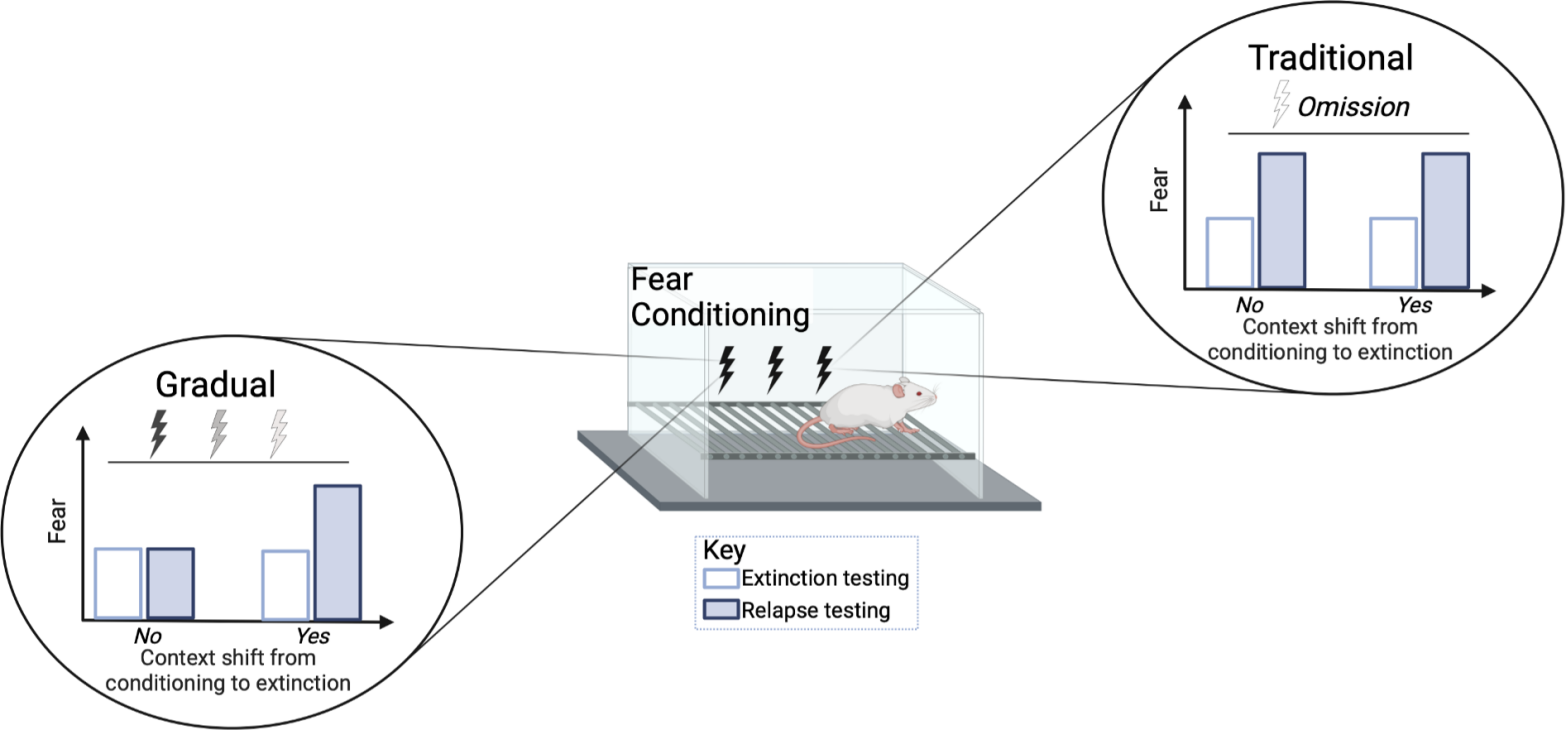
Comparison of standard versus gradual extinction protocols. Kennedy et al. tested two different extinction procedures that reduce learned fear in rats following a classical fear conditioning protocol. The standard or traditional group (right) was exposed to a conditional stimulus (tone) only, without receiving any further electric shocks, while the gradual group (left) received a tone followed by electric shocks of a gradually reducedvoltage. In the traditional group, the fear response was initially reduced (white bar) but increased again during relapse (blue bar); this occurred regardless of whether the environmental conditions were the same (marked as no) or different (marked as yes) between extinction and conditioning. However, gradual extinction reduced relapse, but only when the environmental conditions between conditioning and extinction remained similar.

Collectively, these results add to a growing literature that suggests manipulating the intensity of an unconditional stimulus (such as an electric shock) following training can have a substantial advantage over standard extinction treatments in reducing fear long-term. Kennedy et al. provide a clear explanation for these results that supports recent learning theories: too much variation between the original training shock and what occurs during extinction (as experienced in the standard group) is more likely to create a new inhibitory memory. On the other hand, a smaller change (as is experienced in the gradual extinction group) is more likely to weaken aspects of the original memory and is thus able to reduce fear long term.

These results present a promising first step in increasing the efficacy of extinction-based treatments. A next step will be to test the neural mechanisms that contribute to this effect to determine if and how this process differs from standard extinction practices, and if it could in fact be more similar to other processes relevant for updating memory, such as reconsolidation.
